# Comment on Wardzinski et al. Mobile Phone Radiation Deflects Brain Energy Homeostasis and Prompts Human Food Ingestion. *Nutrients* 2022, *14*, 339

**DOI:** 10.3390/nu14142948

**Published:** 2022-07-19

**Authors:** Michael Witthöft, Ferenc Köteles, Renáta Szemerszky

**Affiliations:** 1 Department of Clinical Psychology, Psychotherapy and Experimental Psychopathology, Johannes Gutenberg-University Mainz, 55122 Mainz, Germany; 2 Institute of Health Promotion and Sport Sciences, ELTE Eötvös Loránd University, 1053 Budapest, Hungary; koteles.ferenc@ppk.elte.hu (F.K.); szemerszky.renata@ppk.elte.hu (R.S.)

Wardzinski and colleagues present the findings of an experimental provocation study, in which the effect of a 25 min exposure to radiofrequency electromagnetic field (EMF) emitted by a mobile phone on food consumption is studied [1]. The authors concluded that EMF exposure is causally related to an increased calorie intake, and speculated that EMF may contribute to the worldwide obesity epidemic. In our view, methodological, theoretical and ethical issues preclude such an unbalanced interpretation.

Methodologically, the small sample size (*n* = 15; all men) represented a major limitation that remained unmentioned and precluded the generalization of the findings. No information was provided regarding the initial sample size calculation and a possible recruitment stopping rule.

A further major limitation represented the unbalanced experimental within-subject design, in which two exposure conditions were compared to one sham exposure condition. Thus, the probability of obtaining an exposure condition as the first condition was twice as likely compared to obtaining the sham exposure condition first. Given this unbalanced design, order effects (i.e., consuming more calories during the first testing, in which the calorie consumption task was completely unexpected) could fully explain the results.

Third, no manipulation check was reported to demonstrate that participants were really blind to the experimental conditions. This is particularly relevant as a single-blinded design was used; thus, the experimenter could have influenced participants’ beliefs. Additionally, the heat emitted by the operating mobile phones and/or generated by the EMF in the tissues of skin could have been informative regarding the actual condition. It would have been necessary to provide a masking heat stimulus [2] during the sham exposure in order to attribute the observed effect to the EMF itself.

From a theoretical perspective, a radiofrequency EMF cannot penetrate the entire adult brain [3]. Thus, it is implausible that exposure impacting mainly the right temporal region can increase the metabolism of another region (the motor cortex), leading to such an increase in calorie consumption. This is further supported by the fact that although the difference between the phones in terms of exposure was marked (0.97 W/kg vs. 1.33 W/kg), the measured metabolic changes did not differ. The major regulatory center of eating behavior, the hypothalamus, was not directly exposed, and physiological indicators of glucose metabolism did not change. The impact of external cues and learned associations on eating behavior is well-known [4]; such factors might have played a more dominant role in participants’ calorie intake.

In summary, the far-reaching claims of the authors require a more careful and comprehensive investigation, including an adequately powered, double-blinded and preregistered experimental study. The authors considered their findings as “alarming”. From an ethical perspective, this uncritical presentation of severely limited findings bears the risk of producing misleading media reports and further provoking nocebo effects among vulnerable people [5].
